# Intervention to Improve Diarrhea-Related Knowledge and Practices Among Informal Healthcare Providers in Slums of Kolkata

**DOI:** 10.1093/infdis/jiab499

**Published:** 2021-10-27

**Authors:** Tanmay Mahapatra, Sanchita Mahapatra, Nandini Datta Chakraborty, Aparna Raj, Bhawani Bakshi, Barnali Banerjee, Snehasish Saha, Abhijit Guha, Shanta Dutta, Suman Kanungo

**Affiliations:** 1 Indian Council of Medical Research-National Institute of Cholera and Enteric Diseases, Kolkata, India; 2 CARE India Solutions for Sustainable Development, Patna, India

**Keywords:** diarrhea, intervention, informal providers, rationality, effectiveness

## Abstract

**Background:**

In the densely populated slums of Kolkata, informal healthcare providers’ (IHP) diarrhea-related knowledge and rationality of practices should be improved to reduce risk of adverse outcome, expenditure, and antimicrobial resistance.

**Methods:**

A multicomponent intervention was conducted among 140 representative IHPs in the slums of 8 wards in Kolkata to assess its impact on their diarrhea-related knowledge and practice. Six intervention modules in local languages were provided (1 per month) with baseline (N = 140) and postintervention (N = 124) evaluation.

**Results:**

Mean overall (61.1 to 69.3; *P* < .0001) and domain-specific knowledge scores for etiology/spread (5.4 to 8.1; *P* < .0001), management (6.4 to 7.2; *P* < .0001), and oral rehydration solution ([ORS] 5.7 to 6.5; *P* < .0001) increased significantly (at α = 0.05) after intervention and were well retained. Impact on knowledge regarding etiology/spread (adjusted odds ratio [aOR] = 5.6; *P* < .0001), cholera (aOR = 2.0; *P* = .0041), management (aOR = 3.1; *P* < .0001), ORS (aOR = 2.3; *P* = .0008), and overall (aOR = 4.3; *P* < .0001) were significant. Intervention worked better for IHPs who practiced for ≥10 years (aOR = 3.2; *P* < .0001), untrained IHPs (aOR = 4.8; *P* < .0001), and pharmacists (aOR = 8.3; *P* < .0001). Irrational practices like empirical antibiotic use for every cholera case (aOR = 0.3; *P* < .0001) and investigation for every diarrhea case (aOR = 0.4; *P* = .0003) were reduced. Rationality of testing (aOR = 4.2; *P* < .0001) and antibiotic use (aOR = 1.8; *P* = .0487) improved.

**Conclusions:**

Multicomponent educational intervention resulted in sustainable improvement in diarrhea-related knowledge and practices among IHPs in slums of Kolkata. Policy implications should be advocated along with implementation and scale-up.

As policy and programmatic focus intensifies on the management of noncommunicable diseases worldwide [1, 2], developing nations, such as India, must not lose momentum in the mitigation of communicable diseases including diarrhea [3]. Despite being preventable and easily treatable, globally, diarrheal diseases remained the second most common cause of mortality and major cause of malnutrition among children under 5 years old [4], the eighth leading cause of mortality overall, and a critical public health concern in low- and middle-income countries [5]. In India, 13% of annual deaths for children under 5 years old are attributable to diarrhea [6].

Because of poor sanitation and water supply, informal settlers such as urban slum-dwellers remain particularly vulnerable [5]. The United Nations defines slums as urban areas marked by issues such as the following: dilapidated housing, overcrowding, lack of easy access to safe water, inadequate sanitation, and uncertain housing tenure [7]. Based on differing definitional criteria in surveys, approximately 65 [8] to 120 million [9] Indians reside in “urban slums”. Extreme poverty and oversight by healthcare programs often culminate into poorer access to quality healthcare, which often results in poorer health outcomes among slum dwellers, compared with even the rural populations [10].

The issue of limited access to health services is also exacerbated by the general health-seeking behaviors of slum residents [11–13], especially for common illnesses such as diarrhea, which is commonly misperceived as mild and nonfatal [14]. These vulnerabilities and challenges in urban slums often coalesce into a situation in which informal healthcare providers ([IHPs] typically without any formal or informal training), practitioners of alternative modalities, and pharmacists become the preferred choice for many slum dwellers [15]. Furthermore, despite standard guidelines, knowledge regarding management of diarrheal diseases remains inadequate even among trained IHPs and more so among IHPs in slum settings [16–21]. This can lead to unnecessary, inadequate, and incomplete dosage of antibiotic therapy, potentially contributing to antibiotic resistance and other health issues [22–24], unnecessary out-of-pocket expenditure [25, 26], inappropriate testing, and increased morbidity and mortality. Therefore, it is imperative that not only should general measures be taken to mitigate issues related to diarrhea management but also efforts that are targeted toward more vulnerable slum populations that predominantly interact with IHPs for primary healthcare. Studies show that interventions aimed at improving knowledge and practice of IHPs through training and assessments can positively impact case management in low-resource settings [27, 28]. An assessment of knowledge and practice of diarrheal management guidelines was thus undertaken among formal and informal providers in slums of Kolkata city, West Bengal, India [16]. It was revealed that a majority of slum residents were being treated by IHPs who have low awareness of treatment protocols, prevention, and control strategies.

We tested a multicomponent educational intervention to increase awareness of these concepts among the nested sample of IHPs used in the preintervention assessment. In this study, we discuss the impact of this intervention on selected knowledge, practice measures, as well as their rationality.

## METHODS

### Study Design and Intervention

The study was designed to assess the impact of a multicomponent educational intervention for improving diarrhea and its management-related knowledge and practice (including rationality of antibiotic use) by IHPs in the urban slums of Kolkata, the third most populous metropolitan city of India. The intervention, spanning a period of 6 months, was implemented in the slum areas of 8 purposively (those having larger sum areas adjacent to each other) selected (from a list of all 141), densely populated, administrative wards (28, 29, 30, 32, 33, 34, 59, and 66) of the Kolkata municipal area in West Bengal state in eastern India.

One hundred forty consenting IHPs who prescribe allopathic medicines to patients with diarrhea were asked to participate in the study. They were randomly selected from an exhaustive list of all pharmacists, alternative medicine-trained practitioners, or unqualified practitioners who practiced in the selected slums for at least the last 6 months and participated in a situation analysis conducted earlier (described elsewhere) [20]. The baseline assessment comprised a preintervention interview of IHPs by trained field investigators using a prevalidated (during the situation analysis) structured tool to determine their overall and domain-wise (symptoms, etiology/spread, cholera, management, and oral rehydration solution [ORS]), diarrhea-related knowledge levels and management practices (fluid management, laboratory investigations, antibiotic use, and rationality thereof). During next 6 months, 1 training module was provided to each participant monthly for 6 months. Two and eight months after the completion of the intervention, the same knowledge and practice questionnaire was readministered to ensure that participants understand the changes and their sustainability if any.

### Training Modules, Assessment Measures, and Supportive Facilitation

The lessons learned from the preintervention assessment were used to develop 6 multicomponent printed booklets (modules) for the IHPs to read: Disease Characteristics including Spread, Assessment and Management of dehydration, Treatment of Diarrhea in Children, Nutritional (food and fluid) Management of Diarrhea, Diarrhea in Adults, and Prevention and Control of diarrhea. The modules (trilingual: in English, Hindi, and Bengali) and assessment methodologies (indicator definitions for knowledge, practice, and rationality) were finalized by experts of National Institute of Cholera and Enteric Diseases (NICED), Indian Council of Medical Research (ICMR), Kolkata based on standard guidelines, and textbooks and observed antibiotic susceptibility patterns among causative organisms of diarrhea in the study area [31–33].

To ensure ease of use, these modules followed a simplistic yet scientific design for information delivery, pictorial representations of important concepts, recap sections for previous modules, and a final self-assessment quiz at the end of each module. Although the investigators provided monthly modules, they also supported IHPs’ learning through demonstrative instructions for proper use, followed by weekly checks through the remainder of the month to ensure continued usage and assessment. Misplaced modules were replaced, and after satisfactory completion of the whole intervention and self-assessments a certificate of participation from NICED-ICMR was provided.

Rationality of antibiotic use (indication and efficacy optimized with risk of side-effect and resistance), fluid management (indication, type), and laboratory investigations (type, timing, etc) were also determined as per the textbooks, guidelines, and susceptibility patterns [14, 16, 20, 31–34]. Irrationally used antibiotics meant those not indicated (because of poor efficacy, commoner side-effect/resistance, etc; eg, ampicillin in case of acute watery diarrhea) at all or for specific types. Likewise, rationality of intravenous fluid therapy and laboratory testing advice and strategy were established, respectively, based on whether ringer lactate/normal saline (rational) or any other fluid (5% dextrose, dextrose-normal saline, etc: irrational) was used to correct severe dehydration among diarrhea cases, whether stool/rectal swab culture was used as the diagnostic test (rational) or not (irrational), and additionally whether testing was advised before antibiotic administration (rational) or not (irrational).

Based on the resources previously mentioned, for knowledge assessment, component-wise answers were scored (incorrect = 0 and correct = 1), summed, and rescaled within 10, domain-wise and within 100 overall. Scores were next categorized into poor/average/good based on their tertile point-based category boundaries.

### Analysis

Descriptive analyses for IHPs and their patient characteristics were determined as the frequency and proportion with corresponding 95% confidence intervals (CIs). Baseline, 2-month, and 8-month postintervention “knowledge” of the IHPs were assessed as mean scores (compared via paired *t* test), in a scale of 10 for domain-specific and in 100 for overall, proportional distribution across their tertile point-based categories (poor/average/good) with corresponding 95% CIs. Practice across variable categories were determined as proportions/percentages with 95% CIs. Multiple binary and ordinal (where outcome categories were >2 and ordinal; eg, poor/average/good) logistic regression analyses were conducted to assess the impact of the intervention on knowledge and practice measured as adjusted (for IHP characteristics: gender, qualification/training, office type, duration, and year of practice) odds ratios (aORs) with corresponding *P* values, to be interpreted as the odds of being in higher ordered categories as opposed lower ones (cumulated over the lower order categories) during postintervention as opposed to preintervention. Sample size calculation and all statistical analyses were conducted using SAS 9.4. One hundred thirty IHPs were required to achieve 80% power to detect 1% change in the mean knowledge score (assuming a score of 61.9—determined in the situation analysis with a standard deviation of ~2 and matched pair correlation of 0.5) [20] with 95% precision through a prepost comparison, after accounting for a 30% loss to follow-up during intervention [20, 29, 30]. We recruited 140 IHPs, and we anticipated an ~5% further dropout during postintervention assessment.

### Ethics Statement

Before the interviews, details of the study were explained to the IHPs in a language that they understand completely, and voluntary written informed consent statements were obtained from each subject. We maintained confidentiality per the standard national guidelines. Data were securely preserved with confidentiality. The study content and procedures were approved by the Scientific Advisory Committee and Institutional Ethics Committee of National Institute of Cholera and Enteric Diseases, Kolkata (No. A-1/2015-IEC).

## RESULTS

Of 140 recruited IHPS, during the intervention, due to migration, sickness, or death, 16 IHPs were lost to follow-up. Hence, 124 baseline IHPs were interviewed, and all module recipient IHPs were interviewed at post intervention (end-line) and included in the analysis. The majority were male (96%), trained in alternative medicine (53%), had a fixed clinic/office (60%), and practiced for 10 years or more (65%). More than half of them were treating patients who belonged to the low-income group (63%), had low-level of diarrhea-related knowledge (69%), presented with some level of severity (56%), and aged more than 15 years (57%) (Table 1).

**Table 1.** Distribution of Individual Characteristics, Patient Type, and Knowledge About Diarrhea Among Informal Healthcare Providers in Kolkata at Baseline (N = 140)

Provider’s Individual Characteristics, Characteristics of Their Patients, and their Knowledge About Diarrhea				Baseline	
Domains	Components		Categories	Frequency (n)	Proportion (95% Confidence Interval)
Practitioners’ Characteristics		Gender	Female	5	3.57 (0.46–6.68)
			Male	135	96.43 (93.32–99.54)
		Qualification/Training	Alternative Medicine	79	56.43 (48.11–64.74)
			No such	61	43.57 (35.26–51.89)
		Office Type	Fixed clinic/office	86	61.43 (53.27–69.59)
			Has no fixed clinic/office	11	7.86 (3.34–12.37)
			Pharmacy	43	30.71 (22.98–38.45)
		Years of Practice	≥10 years	91	65.00 (57.00–73.00)
			5–9 years	36	25.71 (18.38–33.04)
			<5 years	13	9.29 (4.42–14.15)
General Characteristics of the Patients Treated by Individual Practitioners		(a) Regarding socioeconomic status	Very low income	22	15.71 (9.61–21.82)
			Low income	85	60.71 (52.52–68.90)
			Middle income	33	23.57 (16.45–30.69)
		(b) Regarding knowledge about diarrhea	Very low	42	30.00 (22.31–37.69)
			Low	83	59.29 (51.05–67.52)
			Good	15	10.71 (5.53–15.90)
		(c) Regarding severity of presentation	Severe	24	17.14 (10.82–23.46)
			Some	71	50.71 (42.33–59.10)
			Mild/No	45	32.14 (24.31–39.98)
		(d) Regarding average age	<5 years	39	27.86 (20.34–35.38)
			5–15 years	27	19.29 (12.67–25.90)
			More than 15 years	74	52.86 (44.49–61.23)
Knowledge Regarding Diarrhea and Its Management	Domain-Specific Knowledge Level Regarding Diarrheal Diseases and Their Management	Symptoms of diarrheal diseases	Poor	35	25.00 (17.74–32.26)
			Average	69	49.29 (40.90–57.67)
			Good	36	25.71 (18.38–33.04)
		Etiology and spread of diarrheal diseases	Poor	90	64.29 (56.25–72.32)
			Average	20	14.29 (8.42–20.15)
			Good	30	21.43 (14.55–28.31)
		Cholera	Poor	77	55.00 (46.66–63.34)
			Average	32	22.86 (15.82–29.90)
			Good	31	22.14 (15.18–29.11)
		Management of diarrheal diseases	Poor	64	45.71 (37.36–54.07)
			Average	61	43.57 (35.26–51.89)
			Good	15	10.71 (5.53–15.90)
		Oral rehydration solution	Poor	85	60.71 (52.52–68.90)
			Average	25	17.86 (11.43–24.28)
			Good	30	21.43 (14.55–28.31)
	Overall Knowledge Level		Poor	70	50.00 (41.61–58.39)
			Average	53	37.86 (29.72–45.99)
			Good	17	12.14 (6.67–17.62)

**Figure 1.**
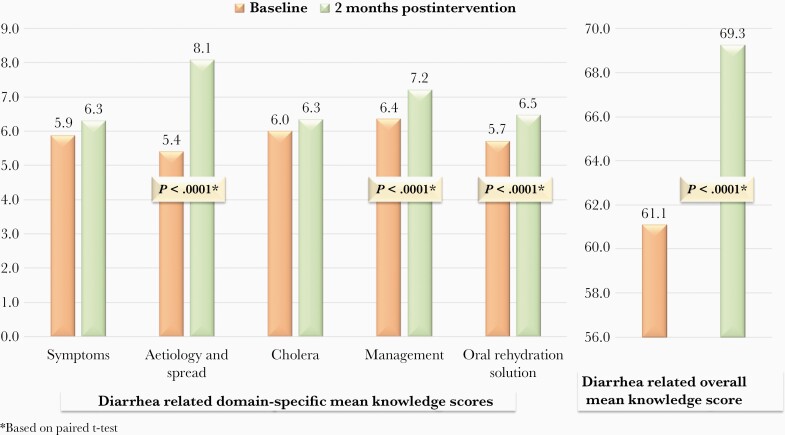
Comparison of overall and domain-specific mean knowledge scores before (N = 140) and 2 months after (N = 124) the intervention.

Regarding IHPs’ mean knowledge scores, significant improvements (*P* < .0001) were observed in overall knowledge (postintervention 69.3 vs baseline 61.1) as well as in the domains of etiology and spread (postintervention 8.1 vs baseline 5.4), management (postintervention 7.2 vs baseline 6.4), and ORS (postintervention 6.5 vs baseline 5.7) (Figure 1).

No significant reduction in the domain-wise and overall mean knowledge scores between 2- and 8-month postintervention assessments indicated retention of knowledge among IHPs once imparted. Improvement even continued to happen for cholera and ORS (Supplementary Table 1).

Upon analyzing the knowledge categories, we observed that before intervention, only one quarter had good knowledge about diarrheal symptoms (26%), but less than that for etiology/spread (21%), cholera (22%), and ORS (21%), and much less for management (11%). Overall baseline knowledge regarding diarrhea was poor among half of them, average for 38%, and good only among 12%. After intervention, the proportions having good knowledge improved substantially for cholera (34% vs 22%) and ORS (28% vs 21%), it doubled for management (20% vs 11%), became ~2.5 times for etiology/spread (54% vs 21%), and was more than 3 times overall (41% vs 12%) (Table 1).

Regarding antibiotic use, disease severity seemed to be the principal driver and proportion of IHPs considering that this increased marginally (76% vs 71%) after intervention. Prescribing an antibiotic empirically to every cholera patient was reduced significantly (52% vs 79%). A huge increase was noted in the use of Isolyte-M by IHPs for correction of severe dehydration (51% vs <1%). Laboratory investigations were reduced from more than one half to less than one third, and among them the proportion that suggested rectal swab culture nearly tripled (76% vs 31%). While managing diarrheal cases by intervening IHPs, a substantial improvement in the rationality of laboratory investigation strategy (timing: 87% vs 47%), overall diagnostic approach (76% vs 46%), antibiotic use (80% vs 68%), and fluid management (76% vs 71%) was observed (Table 2).

**Table 2.** Comparative Distribution of Diarrheal Management Practices Among Informal Healthcare Providers in Kolkata and Their Rationality at Baseline (N = 140) and 2 Months After Intervention (N = 124)

Diarrheal Management Practices and Their Rationality				Baseline		2 Months Postintervention	
Domains	Components		Categories	Frequency (n)	Proportion (95% CI)	Frequency (n)	Proportion (95% CI)
Practice	Most important factor while prescribing antibiotic to diarrhea cases		Severity of disease	99	70.71 (63.08–78.35)	94	76.42 (68.82–84.03)
			Age of the patients	32	22.86 (15.82–29.90)	25	20.33 (13.11–27.54)
			Patients’ affordability	6	4.29 (0.89–7.68)	-	-
			Patients’ preference	3	2.14 (0.00–4.57)	4	3.25 (0.07–6.43)
	Prescribes antibiotics to every diarrhea patient		No	45	32.14 (24.31–39.98)	33	26.83 (18.89–34.77)
			Yes	95	67.86 (60.03–75.69)	90	73.17 (65.23–81.11)
	Prescribes antibiotics to every cholera patient		No	29	20.71 (13.92–27.51)	59	47.97 (39.01–56.92)
			Yes	111	79.29 (72.49–86.08)	64	52.03 (43.08–60.99)
	Usually prescribed IV fluid to treat cases of diarrhea with severe dehydration		5% Dextrose	17	12.14 (6.67–17.62)	2	1.65 (0.00–3.96)
			DNS	24	17.14 (10.82–23.46)	27	22.31 (14.79–29.84)
			Ringer lactate	14	10.00 (4.97–15.03)	6	4.96 (1.04–8.88)
			Normal saline	30	21.43 (14.55–28.31)	24	19.83 (12.63–27.04)
			Isolyte-M	1	0.71 (0.00–2.13)	62	51.24 (42.21–60.27)
			Others	54	38.57 (30.41–46.73)	-	-
	Advise laboratory investigation to identify the causative agent of diarrheal cases		No	61	43.57 (35.26–51.89)	85	69.67 (61.40–77.95)
			Yes	79	56.43 (48.11–64.74)	37	30.33 (22.05–38.60)
	Advised laboratory investigation to identify the causative agent of diarrheal cases		Blood culture	1	0.71 (0.00–2.13)	-	-
			Stool/rectal swab culture	43	30.71 (22.98–38.45)	93	76.23 (68.57–83.89)
			Stool for routine microscopy	77	55.00 (46.66–63.34)	14	11.48 (5.74–17.21)
			Others	19	13.57 (7.83–19.32)	15	12.30 (6.38–18.21)
Rationality of diarrheal management	Rationality of antibiotic use in case of	Acute watery and bloody diarrhea	Irrational	20	14.29 (8.42–20.15)	14	11.57 (5.79–17.35)
			Rational	120	85.71 (79.85–91.58)	107	88.43 (82.65–94.21)
		Mucoid diarrhea	Irrational	32	22.86 (15.82–29.90)	13	10.74 (5.15–16.34)
			Rational	108	77.14 (70.10–84.18)	108	89.26 (83.66–94.85)
		diarrheal diseases as a whole	Irrational	45	32.14 (24.31–39.98)	24	19.83 (12.63–27.04)
			Rational	95	67.86 (60.03–75.69)	97	80.17 (72.96–87.37)
	Rationality of fluid management of diarrhea cases		Irrational	41	29.29 (21.65–36.92)	29	23.97 (16.25–31.68)
			Rational	99	70.71 (63.08–78.35)	92	76.03 (68.32–83.75)
	Advised laboratory investigation to identify the causative agent of diarrheal cases		Irrational	20	14.29 (8.42–20.15)	15	12.30 (6.38–18.21)
			Rational	120	85.71 (79.85–91.58)	107	87.70 (81.79–93.62)
	Strategy (timing) for laboratory investigation to identify the causative agent of diarrheal cases		Irrational	74	52.86 (44.49–61.23)	16	13.22 (7.10–19.35)
			Rational	66	47.14 (38.77–55.51)	105	86.78 (80.65–92.90)
	Overall laboratory diagnostic approach to identify the causative agent of diarrheal cases		Irrational	75	53.57 (45.21–61.94)	29	23.97 (16.25–31.68)
			Rational	65	46.43 (38.06–54.79)	92	76.03 (68.32–83.75)

Abbreviations: CI, confidence interval; DNS, dextrose normal saline; IV, intravenous.

Multiple logistic regression findings (adjusted for IHP characteristics) did not reveal any overall or IHP type-specific change in patient characteristics other than significant improvement in the knowledge (aOR = 1.7, *P* = .0431) of the treated patients in general (Table 3).

**Table 3.** Association of the intervention with change in general characteristics of the patients treated by individual informal healthcare providers in Kolkata (N = 124)

Impact of Intervention on the Patient Characteristics			Higher Age		More Severe Presentation	
Overall Sample			aOR	*P* Value	aOR	*P* Value
			1.48	.1098	1.43	.1535
Across the strata of^a^	Gender	Male	1.45	.1401	1.43	.1640
	Qualification/ Training	Alternative Medicine	1.50	.1834	1.68	.0944
		No such	1.53	.2889	1.35	.4240
	Office Type	Fixed clinic/office	1.37	.2814	1.48	.1910
		Pharmacy	2.26	.0747	1.36	.4750
	Duration of practice	≥ 10 years	1.39	.2343	1.66	.0745
		<10 years	1.74	.2515	1.29	.5759
			Better SES		Better Knowledge	
Overall Sample^b^			aOR	*P* Value	aOR	*P* Value
			1.33	.2632	**1.70**	**.0431**
Across the strata of ^a^	Gender	Male	1.39	.2108	**1.69**	**.0498**
	Qualification/ Training	Alternative Medicine	1.72	.0991	1.33	.3759
		No such	0.93	.8564	2.23	.0628
	Office Type	Fixed clinic/office	1.81	.0788	1.44	.2569
		Pharmacy	1.00	1.0000	2.18	.0953
	Duration of practice	≥ 10 years	1.35	.3062	1.52	.1560
		<10 years	1.35	.5334	1.89	.2197

Boldfaced figures indicate that association is considered to be statistically significant as *P* < .05.

Abbreviations: aOR, adjusted odds ratio; SES, socioeconomic status.

a aOR across the strata of characteristics of the informal healthcare providers are adjusted for the rest of their characteristics.

b aOR in overall sample adjusted for the characteristics of the informal healthcare providers.

In the overall sample, intervention was associated with significant improvement in the IHP’s diarrhea-related overall (aOR = 4.3, *P* < .0001) and domain-specific knowledge regarding etiology/spread (aOR = 5.6, *P* < .0001), cholera (aOR = 2, *P* = .0041), management (aOR = 3.1, *P* < .0001), and ORS (aOR = 2.3, *P* = .0008).

For etiology/spread and overall knowledge, significant association with improvements were noted in each strata of IHP type and their practice duration. For knowledge regarding diarrheal symptoms and cholera, intervention was significantly beneficial for only pharmacy-based (aOR = 2.4, *P* = .0312) and less (practicing <10years) experienced (aOR = 2.6, *P* = .0408) IHPs, respectively. Except for fixed clinic-based and more experienced IHPs, intervention improved knowledge regarding management of diarrhea among all others, whereas among more experienced (practicing for 10 years or more), improvement in knowledge about ORS was associated (aOR = 1.7, *P* = .0469) positively with intervention (Table 4).

**Table 4.** Association between intervention and better domain-specific and overall knowledge regarding diarrhea and its management among informal healthcare providers in Kolkata (N = 124)

Impact of Intervention (Reference: Preintervention) as Odds of Improvement			In Diarrhea-Related Knowledge Level Regarding					
			Symptoms		Aetiology and Spread		Cholera	
In Overall Sample^a^			aOR	*P* Value	aOR	*P* Value	aOR	*P* Value
			1.29	.2856	**5.60**	**<.0001**	**2.01**	**.0041**
Across the strata of^b^	Qualification/Training	Alternative Medicine	0.65	.1333	**2.31**	**.0050**	1.32	.3373
		No such	1.58	.1955	**16.01**	**<.0001**	1.81	.1178
	Office type	Fixed clinic/office	0.6	.0712	**2.63**	**.0009**	1.54	.1260
		Pharmacy	**2.44**	**.0312**	**23.23**	**<.0001**	1.52	.3271
	Duration of practice	≥10 years	1.08	.7731	**3.93**	**<.0001**	1.25	.4049
		<10 years	0.62	.2579	**4.43**	**.0010**	**2.61**	**.0408**
			Management		ORS		Overall	
In Overall Sample^a^			aOR	*P* Value	aOR	*P* Value	aOR	*P* Value
			**3.09**	**<.0001**	**2.28**	**.0008**	**4.31**	**<.0001**
Across the strata of^b^	Qualification/Training	Alternative Medicine	**1.82**	**.0443**	1.67	.0870	**2.26**	**.0052**
		No such	**2.83**	**.0052**	2.00	.0551	**4.75**	**<.0001**
	Office type	Fixed clinic/office	1.63	.0863	1.60	.1035	**2.00**	**.0138**
		Pharmacy	**4.95**	**.0006**	2.21	.0570	**8.27**	**<.0001**
	Duration of practice	≥10 years	**2.29**	**.0028**	**1.70**	**.0469**	**3.15**	**<.0001**
		<10 years	1.92	.1266	2.40	.0691	**2.43**	**.0380**

Boldfaced figure indicates that association is considered to be statistically significant as *P* < .05.

Abbreviations: aOR, adjusted odds ratio; ORS, oral rehydration solution.

a aOR in overall sample adjusted for the characteristics of the informal healthcare providers.

b aOR across the strata of characteristics of the informal healthcare providers are adjusted for the rest of their characteristics.

Odds of laboratory investigation in each diarrhea case (aOR_overall_=0.38, *P* = .0003) and antibiotic use in each cholera case (aOR_overall_=0.29, *P* < .0001) decreased significantly upon intervention in whole sample and in each stratum. Intervention improved rationality of antibiotic use in the whole sample as well as among pharmacy-based IHPs for overall diarrheal diseases (aOR_overall_=1.81, *P* = 0487, aOR_overall_=6.53, *P* = .0198) and specifically for mucoid type (aOR_overall_=2.34, *P* = 0203, aOR_overall_=8.91, *P* = .0440). Rational use of antibiotics for mucoid diarrhea treatment improved significantly among unqualified (aOR = 3.14, *P* = .0407) and more experienced IHPs (aOR = 2.20, *P* = .0413). Positive deviances were also observed in the rationality of the overall approach (aOR_overall_=4.19, *P* < .0001) and timing strategy (aOR_overall_=8.39, *P* < .0001) for the laboratory investigations among IHPs as a whole, as well as in each stratum (Table 5).

**Table 5:** Association of intervention with diarrheal management practices and their rationality among informal healthcare providers in Kolkata (N = 124)

Impact of Intervention on			Drivers of Antibiotic Use (Reference: Disease Severity)				Prescribing (Reference: Not) Antibiotic to Every			
			Patient\'s Age		Affordability/Preference		Diarrhea Case		Cholera Case	
In Overall Sample^a^			aOR	*P* Value	aOR	*P* Value	aOR	*P* Value	aOR	*P* Value
			0.86	.6240	0.41	.1513	1.36	.2668	**0.29**	**<.0001**
Across the strata of ^b^	Gender	Male	0.85	.6163	0.41	.1505	1.50	.1552	**0.30**	**<.0001**
	Qualification/ Training	Alternative Medicine	0.64	.2823	0.51	.4448	0.96	.9003	**0.24**	**.0003**
		No such	1.11	.8125	0.45	.3521	1.93	.1193	**0.31**	**.0038**
	Office type	Has fixed clinic/office	0.78	.4978	0.36	.3762	1.11	.7598	**0.27**	**.0003**
		Pharmacy	0.75	.5885	0.47	.3118	1.38	.4882	**0.26**	**.0039**
	Duration of practice	≥10 years	0.88	.7313	0.39	.1782	1.27	.4376	**0.37**	**.0017**
		<10 years	0.75	.5785	1.08	.9557	1.59	.4921	**0.11**	**.0005**
			Suggesting (Reference: Not) Some Laboratory Investigation to Each		Rational (Reference: Irrational) Antibiotic Use in Case of					
					Acute Watery and Bloody Diarrhea		Mucoid Diarrhea		Diarrheal Diseases as a Whole	
In Overall Sample^a^			aOR	*P* Value	aOR	*P* Value	aOR	*P* Value	aOR	*P* Value
			**0.38**	**.0003**	1.23	.5980	**2.34**	**.0203**	**1.81**	**.0487**
Across the strata of ^b^	Gender	Male	**0.37**	**.0003**	1.24	.5808	**2.15**	**.0388**	1.70	.0805
	Qualification/ Training	Alternative Medicine	**0.29**	**.0002**	1.18	.7226	2.13	.0653	1.58	.1941
		No such	**0.37**	**.0252**	1.44	.5504	4.41	.0665	**3.14**	**.0407**
	Office type	Has fixed clinic/office	**0.34**	**.0009**	0.74	.4953	1.94	.0940	1.35	.3693
		Pharmacy	**0.24**	**.0063**	6.32	.0949	**8.91**	**.0440**	**6.53**	**.0198**
	Duration of practice	≥10 years	**0.36**	**.0009**	0.93	.8737	**2.20**	**.0413**	1.62	.1414
		<10 years	**0.28**	**.0098**	3.39	.1454	5.82	.1116	3.92	.0513
			Rational (Reference: Irrational) Fluid Management of Diarrhea Cases		Rational (Reference: Irrational) Lab Test to Identify the Agent		Rational (Reference: Irrational) Strategy (Timing) for Lab Test		Overall Rational Lab Test (Reference: Irrational) Approach	
In Overall Sample^a^			aOR	*P* Value	aOR	*P* Value	aOR	*P* Value	aOR	*P* Value
			1.31	.3496	1.22	.5922	**8.39**	**<.0001**	**4.19**	**<.0001**
Across the strata of ^b^	Gender	Male	1.29	.3964	1.15	.7231	**8.29**	**<.0001**	**4.13**	**<.0001**
	Qualification/ Training	Alternative Medicine	1.45	.2828	1.27	.6445	**6.35**	**<.0001**	**3.59**	**.0004**
		No such	1.09	.8678	1.12	.8314	**9.17**	**<.0001**	**3.81**	**.0015**
	Attachment	Has fixed clinic/office	1.29	.4537	1.23	.6408	**7.61**	**<.0001**	**3.45**	**.0003**
		Pharmacist	1.60	.4472	0.72	.6408	**8.05**	**.0002**	**4.35**	**.0026**
	Duration of practice	≥10 years	1.41	.2774	0.95	.8980	**6.77**	**<.0001**	**3.14**	**.0003**
		<10 years	1.02	.9717	2.19	.2850	**9.17**	**.0004**	**5.70**	**.0016**

Boldfaced figures indicate that association is considered to be statistically significant as *P* < .05.

Abbreviations: aOR, adjusted odds ratio; Lab, laboratory.

a aOR in overall sample adjusted for the characteristics of the informal healthcare providers.

b aOR across the strata of characteristics of the informal healthcare providers are adjusted for the rest of their characteristics.

## DISCUSSION

Unqualified and unregulated healthcare providers (including pharmacists) identified in this study as IHPs are often deeply integrated in rural and urban slum communities. Current investigation highlighted the potential for tailored intervention to bridge the preidentified gaps in their diarrhea-related knowledge and practice including rational use of antibiotics. It appeared that improvement of their overall knowledge is fast, practice is also knowledge-driven, but development of theoretical symptomatologic knowledge and indication of antibiotic use are relatively difficult as opposed to practical knowledge for understanding the etiology/spread, treating cases of diarrhea, cholera, administering ORS, etc. Translation of the knowledge into practice is also more difficult for use of intravenous fluids, laboratory investigations, and their timing (preantibiotic use), whereas improvement in the rest of the practices is relatively fast as per expectation.

These enterprising, accessible, and responsive IHPs provide affordable services. Therefore, in these less than ideal, remote or less-privileged settings, more than 70% of primary healthcare needs are fulfilled by them [15, 35–38]. Given that IHPs remained the preferred providers for treatment of common illnesses among vulnerable populations, simple, scalable, and expeditious interventions aimed at improving awareness regarding the disease, its prevention, and management guidelines are urgently needed among IHPs to reduce incidences of case mismanagement and consequent adverse outcomes [39, 40]. Furthermore, their roles in the community and circumstantial popularity can be leveraged to promote prevention and control strategies that lie outside the ambit of medical practice, as evidenced by reports from other developing countries as well as India. [28, 41–43]. Appropriate management of diarrheal diseases was chosen as the focus of this intervention because conditions characterizing slum settings such as poor personal hygiene, water access, and sanitation and improper waste disposal are all common risk factors for diarrhea, especially among children under 5 years old [6]. However, even though diarrheal diseases pose a significant public health concern, diarrhea is an easily preventable, treatable, and often self-limiting condition. In particular, diarrhea-related mortality and morbidity due to dehydration and malnutrition can be prevented in an overwhelming number of cases with appropriate administration of ORS and nutrition management. More importantly, the World Health Organization does not recommend the routine use of antimicrobials or antidiarrheal drugs, especially among children [44]. Although international and contextualized treatment protocols [45] have been clearly outlined, awareness and adherence to the same remain low even among trained providers [17–19]. In the preintervention assessment conducted prior this study, as expected, the knowledge and practice measures were lower for IHPs compared with qualified physicians [16]. Therefore, it seemed important to orient IHPs on these guidelines and discourage unnecessary medical interventions, testing, and irrational antibiotic use [20]. The educational intervention used in this study was developed after the initial assessment of the selected IHPs, such that the training modules provided later were tailored to address gaps and misperceptions among them. The intervention significantly increased knowledge scores among IHPs for important subdomains of etiology/spread and management of diarrheal diseases at 2 months postintervention. These scores declined marginally at 8 months postintervention but remained significantly higher than preintervention scores, indicating meaningful retention of training content. Even more encouraging results were seen for knowledge of ORS, which showed an increase at both postintervention and retention assessment timepoints. Likewise, overall knowledge scores increased after the intervention and did not show any decline when retention was assessed. When knowledge scores were categorized as “poor”, “average”, and “good” based on their tertile boundaries, for a significant proportion of IHPs, knowledge level improved from poor or average categories to good after the intervention. Similar improvements were seen across the aforementioned subdomains as well as for cholera. The results further indicated that improvement in knowledge translated into better practice among providers. An increased proportion of IHPs prescribed antibiotics rationally for mucoid diarrhea as well as for diarrhea as a whole. Fewer IHPs were prescribing antibiotics empirically for every cholera patient, and this number substantially decreased at 8 months postintervention. The same decreasing trend was seen for advice of laboratory investigations in every diarrhea case to identify causative organisms. The strategy for testing also became more rational in terms of timing, as proportions suggesting laboratory investigation after starting antibiotic reduced significantly. Furthermore, we observed that as IHPs reduced testing for patients, there was an increase in the number of IHPs choosing more appropriate tests such as stool and/or rectal swab cultures. This suggests that given enough time to assimilate scientific information during a prolonged intervention, IHPs can be encouraged to defer to standard treatment guidelines. The proportion of IHPs exercising the overall rational approach for laboratory investigation also increased after intervention. Regression analysis clearly revealed a positive impact of intervention on diarrhea-related knowledge and practice measures among IHPs practicing in these urban slums. Intervention was associated with more than 3 times likelihood of improvement in the overall knowledge levels of the participating IHPs. A similar positive impact of the intervention was noted regarding IHPs’ knowledge about certain subdomains such as etiology and spread of diarrheal disease, cholera, management of diarrheal diseases, and use of ORS. However, for the rationality of choice regarding the intravenous fluid for the management of cases presented with severe dehydration, the impact of the intervention was not significant. Continued intervention may be required to improve these practices significantly.

After analyzing the impact of the intervention across different strata of IHP characteristics, we discovered that the training material was effective for practitioners without any qualification or prior training. However, IHPs with fixed clinics and pharmacists were more responsive to the intervention, whereas no effect was seen for those without any fixed clinics. In addition, the knowledge levels of IHPs with less than 5 years of experience did not seem to notably improve after the intervention. Lesser antibiotic use in cholera and lesser laboratory investigations for identification of causative agents in diarrheal diseases were associated with the intervention for the overall sample of IHPs as well as across almost all strata of provider characteristics. Moreover, rational antibiotic use for diarrheal diseases as a whole and rational laboratory investigation approach also showed positive associations with the intervention. On the other hand, without some improvement in diarrhea-related knowledge levels in general, intervention did not result in any significant change in the characteristics of the treated patient pool, such as average age, socioeconomic status, and severity of presentation for the overall sample, as well as across almost all strata of provider characteristics. Therefore, we could assume that changes in knowledge and practice when compared with preintervention measures were not influenced by different patient profiles.

Congruent with these findings, similar community-based and nongovernmental organization-driven health education interventions that have focused on improving knowledge of concepts such as appropriate ORS use among general slum residents [46], those targeting informal or unlicensed providers, and community health volunteers for improving management of childhood illnesses including diarrheal diseases have previously shown encouraging results in other developing countries [47], including our neighbors [48, 49] as well as elsewhere in India [27, 50].

This study had some limitations that should be considered when interpreting the results discussed herein. Because the findings of the study are based on pre- and postintervention comparison, the observed associations may not be interpreted as causal. Any effort to extrapolate the results beyond the study sample should be made with caution. Social desirability bias may potentially be there in the patient characteristics and practice-related data provided by the IHP, although, even then, overall influence on the findings is likely to be miniscule because the analyses were self-matched for IHPs. Furthermore, these interview tools were kept succinct and therefore knowledge and practice measures could not be assessed in further detail.

Despite these limitations, the current study, by virtue of its multicomponent interventional design, strategic implementation plan, strict vigilance on adherence through regular follow-up, and robust analytics, could generate important insights for developing such intuitive, pictorial, yet comprehensive modules to intervene in IHP practices. Given the vulnerabilities prevailing in the urban slums, the observed improvement in diarrhea-related knowledge and management among IHPs (the preferred care-providers for the dwellers in these settings) has immense potential to reduce (1) diarrhea-related morbidity and mortality, especially among children under 5 years old, (2) overall out-of-pocket expenditure, as well as (3) microbial resistance owing to indiscriminate antibiotic use.

## CONCLUSIONS

Diarrhea-related knowledge and case management practices including rationality were much poorer than optimum in the densely populated, resource-poor urban slum settings of Kolkata, India. The high-yield, low-cost effort to improve the knowledge, rationality of laboratory investigations, fluid management, and antibiotics use for the management of diarrheal cases by the practicing IHPs in the urban slums of Kolkata seemed to be impactful and sustainable. Large-scale implementation and concurrent evaluation may be considered as the next scale-up plan to translate the generated evidence to the next course of action and then policy.

## Supplementary Data

Supplementary materials are available at *The Journal of Infectious Diseases* online. Supplementary materials consist of data provided by the author that are published to benefit the reader. The posted materials are not copyedited. The contents of all supplementary data are the sole responsibility of the authors. Questions or messages regarding errors should be addressed to the author.

jiab499_suppl_Supplementary_Materials

jiab499_suppl_Supplementary_Table_S1

## Notes

***Acknowledgments.*** We sincerely acknowledge the support and hard work of the community health workers and field supervisors and other project staff of the ICMR-National Institute of Cholera and Enteric Diseases (NICED) working in the study area in different projects for extending their support and facilitation of the field work. We also thank all the participants and the community involved in the study as well as the concerned authority in the local area and ICMR-NICED for approving and supporting the study.

***Author contributions.*** S. K. and T. M. conceived and designed the study and developed the intervention modules. S. D. and S. K. acquired all approvals for the intervention. T. M., S. M., N. D. C., Ba. B., S. S., and A. G. managed the logistics for the study. T. M., S. M., N. D. C., S. S., and A. G. implemented the study. S. K. supervised the whole implementation process. N. D. C., A. R., Bh. B., S. S., and A. G. collected the data. T. M., S. M., A. R., and Ba. B. analyzed the data. S. K. and T. M. supervised the whole data analysis and interpretation. T. M., S. M., A. R., and Bh. B. drafted the manuscript. S. K. and S. D. critically reviewed and finalized the manuscript. All authors reviewed and approved the final version of the manuscript for submission. S. K. attests that all listed authors meet authorship criteria and that no others meeting the criteria have been omitted.

***Disclaimer.*** The funders played no role in study design, data collection and analysis, decision to publish, or preparation of the manuscript.

***Financial support.*** This work was funded by the Institutional Intramural Funding (IM/SK/12-13/1) of Indian Council of Medical Research-National Institute of Cholera and Enteric Diseases, Kolkata, Government of India.

***Potential conflicts of interest.*** All authors: No reported conflicts of interest. All authors have submitted the ICMJE Form for Disclosure of Potential Conflicts of Interest.

Presented in part: 15th Asian Conference on Diarrhoeal Disease and Nutrition (ASCODD 2020), January 28–30, 2020, Dhaka, Bangladesh.
