# Supplementary material for: Intervention to Improve Diarrhea-Related Knowledge and Practices Among Informal Healthcare Providers in Slums of Kolkata
Source: J Infect Dis. 2021 Oct 27;224(Suppl 7):S890–900. doi: 10.1093/infdis/jiab499 (PMC8687078; doi:10.1093/infdis/jiab499)
Supplement: jiab499_suppl_Supplementary_Materials [file jiab499_suppl_supplementary_materials.docx]

**Supplementary Table S1.** Comparative distribution of domain-wise and overall mean knowledge scores between 2 and 8 months postintervention.
