# Supplementary material for: Intervention to Improve Diarrhea-Related Knowledge and Practices Among Informal Healthcare Providers in Slums of Kolkata
Source: J Infect Dis. 2021 Oct 27;224(Suppl 7):S890–900. doi: 10.1093/infdis/jiab499 (PMC8687078; doi:10.1093/infdis/jiab499)
Supplement: jiab499_suppl_Supplementary_Table_S1 [file jiab499_suppl_supplementary_table_s1.docx]

Table S1. Comparative distribution of domain-wise and overall mean knowledge scores between two and eight months post-intervention

|  |  | **Two months Post-intervention** | | **Eight months Post-intervention** | |
| --- | --- | --- | --- | --- | --- |
|  |  | **N** | **Mean (95%CI*)** | **N** | **Mean (95%CI*)** |
| Diarrhea related domain-specific mean knowledge scores | Symptoms | 123 | 6.30(5.97-6.63) | 110 | 6.11(5.78-6.44) |
|  | Aetiology and spread | 124 | 8.08(7.66-8.50) | 109 | 7.74(7.32-8.17) |
|  | Cholera | 123 | 6.34(6.05-6.63) | 110 | 6.39(6.11-6.67) |
|  | Management | 124 | 7.20(6.93-7.46) | 110 | 7.10(6.85-7.35) |
|  | Oral rehydration solution | 123 | 6.47(6.23-6.70) | 110 | 6.89(6.67-7.11) |
| Overall | | 122 | 69.25(67.66-70.84) | 107 | 70.08(68.40-71.75) |

* 95%CI = 95% Confidence Interval
